# Reinventing Palliative Care Delivery in the Era of COVID-19: How Telemedicine Can Support End of Life Care

**DOI:** 10.1177/1049909120948235

**Published:** 2020-08-07

**Authors:** Katherine C. Ritchey, Alice Foy, Erin McArdel, David A. Gruenewald

**Affiliations:** 1 Palliative Care and Hospice Service, Geriatrics and Extended Care Service, Veterans Affairs Puget Sound Healthcare System, Seattle, WA, USA; 2 Division of Gerontology and Geriatric Medicine, Department of Medicine, University of Washington School of Medicine, Seattle, WA, USA; 3 Chaplain Service, Palliative Care and Hospice Service, Veterans Affairs Puget Sound Healthcare System, Seattle, WA, USA

**Keywords:** telemedicine, end of life, geriatrics, veteran, COVID-19

## Abstract

Telemedicine technology has become essential to healthcare delivery in the COVID-19 era, but concerns remain regarding whether the intimacy and communication that is central to high-quality palliative care will be compromised by the use of this technology. We employed a business model approach to identify the need for system innovation in palliative care, and a quality improvement approach to structure the project. Products from this project included a standard operating procedure for safe use of tablet computers for inpatient palliative care consultations and family visitations; tablet procurement with installation of video telehealth software; and training and education for clinical staff and other stakeholders. We describe a case illustrating the successful use of palliative care telehealth in the care of a COVID-19-positive patient at the end of life. Successful use of video telehealth for palliative care involved overcoming inertia to the development of telehealth infrastructure and learning clinical video telehealth skills; and engaging front-line care staff and family members who were open to a trial of telehealth for communication. Information gleaned from family about the patient as a person helped bedside staff to tailor care toward aspects meaningful to the patient and family and informed best practices to incorporate intimacy into future palliative video consultations and family visit.

## Introduction

In the wake of the coronavirus pandemic, healthcare has suddenly and radically transformed. Healthcare facilities and staff were by patients rapidly deteriorating and dying of COVID-related illness. Skilled nursing facilities have been devastated, accounting for about a quarter of documented deaths from COVID-19 in the United States.^1^ Healthcare workers worldwide have faced unprecedented challenges, including accommodating to the need for physical distancing, supporting families who are prevented from seeing their critically ill loved ones, managing acute bereavement when patients die; and in some cases, working outside their usual scope of practice.^2,3^ Numerous heartbreaking stories have appeared in news media and professional publications about patients dying alone, with their loved ones denied permission to visit.^4-8^

Palliative care has been highlighted as an essential part of the pandemic response.^9^ Among the key roles served by palliative care teams (PCT) are developing system-level pandemic response plans; serving on scarce resource allocation teams; identifying and addressing goals of care; supporting effective symptom management; providing psychosocial support and bereavement care for family members; and supporting other healthcare workers who are overwhelmed, stressed and traumatized by exposure to traumatic events they have witnessed.^10-13^

Telemedicine technology was being used increasingly even before the COVID-19 pandemic,^14^ but the pandemic has vaulted telemedicine into the role of critical healthcare infrastructure.^15^ During this time of social distancing and isolation, restoring meaningful human connection has required creativity and rapid innovation. There has been keen interest in the use of telehealth technology in palliative care, to improve communication between isolated patients and their families, and between patients and their care providers.^8,15-17^

Below, we share highlights of how this paradigm shift was brought to fruition in a single Veterans Affairs Medical Center and how the intimacy necessary for end-of-life care was maintained through the integration of telehealth technology into the work of the PCT. We re-conceptualized the delivery of palliative care services as a business model innovation, to identify and implement the components required for adapting and sustaining high quality care at a time when traditional care was no longer possible, but the need for care was great. Additionally, we structured our innovation using a quality improvement approach adapted from other commonly used models (Table 1).^18-20^

**Table 1.** Framework for Quality Improvement Project.

Identify the problem
Convene workgroup
Define the challenge – baseline status, barriers, facilitators
Analyze the problem What needs to change? clinical practice/system design/work processes; education/training to support new practices; documentation
Develop Action Plan – specific objectives and assignments
Implement Action Plan in a test case
Evaluate and feedback progress
Modify/extend initial Action Plan as needed

### Restructuring the System of Care Delivery

“Business model” is a conceptual framework coined in the mid-1990’s and whose definition has evolved over the past several decades.^21^ Simply defined, a “business model is a description of an organization and how that organization functions in achieving its goals (e.g. profitability, growth, social impact,…)”.^22^ Though healthcare is not a traditional “business,” it may be helpful to apply a “business model” framework when restructuring the healthcare delivery system is necessary. When circumstances threaten the delivery of a service or good, restructuring requires critical appraisal of current functions and awareness that there will be a need for model evolution and/or innovation due to internal or external changes over time.^23^

In times of systemic disruption, innovation is required to overcome barriers and identify new strategies for a business to persevere (e.g. maintain healthcare service delivery). Innovation may be defined as “the process of transforming one ‘business model’ (either entire or core components) to another.”^24^ Sustainable innovation involves analysis and planning with clear stages, actions, and pitfalls and an imperative to overcome inertia to change.^25,26^ (Table 2).

**Table 2.** Change Management Stages, Actions and Pitfalls.^25^

Stage	Examples from our experience with integrating tablets into palliative care
Establish a sense of urgency	Sense of urgency ensured by pandemic, and acknowledged need to prioritize telehealth for clinical care across the healthcare system
Form a powerful guiding coalition	Collaboration with Acute Care Committee and building on long-standing relationships and alliances with medical ward leadership
Create a vision	Need identified from initial review of literature, social media, etc. Brainstorming on uses of tablets, not only for outpatient and home care but also for inpatients and family visitation
Communicate the vision	Stakeholder buy-in elicited in committee meetings and individual outreach
Empower others to act on the vision	Identified a common goal that motivated participants to persevere. Leveraged climate of “get things done quickly” during pandemic, and loosening of restrictions on use of communications platforms such as FaceTime for duration of crisis
Plan for and create short-term wins	Completed successful test calls and encounters with patient-owned devices; completed an encounter with team tablet after SOP approval
Consolidate improvements and produce more change	Create and update a step-by-step guide for video chats; incorporate telehealth encounters as part of provider pay for performance and PCT SMART Goal for FY 2020
Institutionalize new approaches	SOP is on the facility SharePoint website; PCT members and trainees being trained to do telehealth visits Front-line staff being trained how to access tablets and virtual palliative care services

Abbreviations:

SMART – specific, measurable, attainable, relevant, time-bound.

SOP – standard operating procedure.

PCT – palliative care team.

FY – fiscal year.

The prerequisites to innovation include not only knowledge, expertise and experience, but also courage, creativity and fearless leadership. Within misfortune or disappointment, it is often hard to embrace opportunities for growth and transformation. It is natural to hide behind fear, blame circumstances for failure, and resist change.

Finally, innovation requires teamwork. No one person can develop and implement changes in systems or models without engagement of key stakeholders and those who oversee or provide critical aspects of service delivery. Teamwork involves diversity, simulating the innovative design process and fueling creativity for model re-design and implementation.

### Problem Statement

The COVID-19 pandemic posed a threat to essential palliative care services, including building connections between patients, families, and healthcare teams; mitigating isolation, loneliness, and fear; managing symptoms; determining care priorities in the face of life threatening illness; and promoting comfort, connectedness and dignity during the dying process. As in other medical centers, patient visitation was severely limited and was nearly entirely discontinued in the hospital-based skilled nursing facility (“Community Living Center [CLC]”) where our inpatient hospice service resides. Additionally, our ability to perform inpatient palliative care consultations was limited by the need to preserve PPE. Our goal was to find ways to consult with patients, manage symptoms, and connect care providers, patients and families to allow for goals of care discussions, enhance the comfort of the environment, address isolation and loneliness, and minimize collective distress from Veterans “dying alone.”

To identify barriers affecting delivery and quality of palliative care, we reviewed current literature, stories, testimonials and social media to assess the fears, concerns and lived experiences of families, patients and providers on the front lines of the pandemic.^4-8^ This provided a deeper appreciation for how the current pandemic has augmented moral distress surrounding isolation at the end of life (EOL), including inability to make physical contact and say last “good-byes”; lack of funerals or collective celebrations of life or grieving and recognition of the uniqueness of individual lives amid massive loss of life; and the lack of other expressions of human connection, belonging and care. We noted the potential for tablet computers and video conferencing to bridge the isolation gap and support palliative care services, as well as the potential for telehealth visits near the EOL to go terribly wrong.^15-17,27-29^

### Convene a Workgroup

We identified people within the PCT who could commit time and expertise in the following domains: telemedicine, quality improvement, patient care coordination, and EOL and grief support services. As this group developed methods, plans and materials, we kept other PCT members and facility stakeholders informed of our progress.

### Define the Challenge

Before the pandemic, our PCT had taken steps to receive telehealth training and begin video visits for outpatients, but competing institutional priorities delayed palliative care telehealth implementation. As the pandemic unfolded, there was a general embrace of rapid initiation and utilization of telehealth by the PCT, patients, and the entire healthcare system. On March 19, 2020, VA also granted permission for VA clinicians to use alternative non-public-facing video conferencing platforms during the public health emergency which increased the ease and availability of telehealth communications.

The flexibility and availability of tablet computers and multiple video conferencing platforms resolved some initial barriers to telemedicine implementation. However, the processes for coordinating inpatient virtual consultations and family visits along with infection prevention procedures for tablet cleaning had not been specifically described by other VA PCTs or facilities. Thus, we aimed to develop a formal standard operating procedure (SOP) for tablet use for inpatient consultations and quickly train PCT staff to facilitate telehealth encounters between providers, patients and families.

### Analyze the Problem

We initiated discussions with providers across VA and non-VA systems and with local telemedicine champions to assess “best practices” regarding the use of tablets, video chat platforms, and procedures for safely transferring tablets in and out of patient rooms. We referenced internal VA guides for training clinicians in the use of telehealth within VA, and ensuring compliance with VA approved video telecommunications procedures.^30,31^ We reviewed existing protocols to ensure safe disinfection of tablets after use.^32,33^ We also began to consider ways to “humanize” the care process during video chat sessions (Table 3).^29^

**Table 3.** Humanizing Technology for Communication Near the End of Life.

Elements	Specific Steps/Examples
Prepare family prior to the conference with what they may see	If patient is sedated and unconscious, describe the comfort measures provided and how symptoms are being addressed If the patient is on a mechanical ventilator, describe how that will look before starting the visit
Provide regular reassurance and check-ins	Name everyone in the room and how they are connected to the patient Guide the family on what things to say Check-in on emotions Explain how the patient is being monitored
Discuss the “pitfalls of technology”	Set expectations about quality of video/sound/etc. Have a back-up plan if video connection fails Name and address frustrations which may develop due to the use of technology as “not good enough” or “not the same” Consider a “test call” with family prior to visit to make sure technology works on both ends
Consider elements of physical and human contact with front line staff	Sing favorite songs Have family watch elements of gentle/comfort-related care such as moistening lips, holding patient’s hand, etc. Have the family share favorite stories, reminisce about the patient, describe the legacy the patient has created

### Implement an Action Plan

We developed a task list to organize our activities and assign tasks among the workgroup members (Table 4). This included procuring tablets that operated through guest wi-fi services and completing “test calls” confirming our ability to perform telehealth encounters throughout the facility; training PCT members in the use of VA and non-VA video conferencing platforms; and developing a facility-wide standard operating procedure (SOP) for the safe use of tablet computers for clinical video telehealth consultations and patient-family visitations for both inpatient Palliative Care Services and CLC Veterans (Supplemental Table 1).

**Table 4.** Initial Task List.

Discuss with local telehealth champions and obtain examples of best practices for use of tablets for clinical patient care
Ensure PCT members have received clinical video telehealth training
Obtain tablet computers
Set up tablets with audio/video communication software
Perform trial to establish communication link between tablets on facility wi-fi network
Develop SOP based on current best practices and submit to facility for approval
Provide training and education for clinical staff to use tablets according to the SOP
Identify plan for storage and tracking use of tablets as part of SOP

We then engaged with stakeholders across our facility to inform and educate about our PCT service adaptations to incorporate telehealth into inpatient EOL care. Our stakeholders included local telemedicine champions, medical directors and nurse managers of Intensive Care Units and COVID-Care Units; facility leadership and administrators; members of Infection Control, Acute Care, and Critical Care Committees; and leaders in Hospital/Specialty Medicine, Geriatrics/Extended Care, CLC, Mental Health, Chaplaincy, and the Emergency Department. We implemented COVID-specific training for bedside nurses on palliative communication strategies, EOL care, and utilization of technology modalities; facility-wide sharing of our SOP; and meetings with medical directors and RN managers of COVID-specific and intensive care units regarding how to access tablets and virtual EOL support services from the PCT. After these measures were in place, we trialed our care approach in the case described below.

### Case of Mr. L

Mr. L was a 71-year-old man with type 2 diabetes mellitus who was admitted to the VA with COVID-19 pneumonia after leaving Hospital A against medical advice. He was initially admitted to Hospital A on April 29th, 2020 with fatigue and poor oral intake. A COVID-PCR test obtained at admission was positive. His hospital course was complicated by superimposed bacterial pneumonia, delirium, malnutrition, and acute kidney injury. His family felt he was not receiving adequate care at Hospital A. They felt nurses were rarely going into his room and they worried about his isolation and not receiving human touch. One of his family members risked exposure to COVID to take Mr. L out of Hospital A and bring him to the VA where they hoped he would receive care more to their liking.

After VA admission on May 7th, 2020, his course was complicated by agitated delirium. He became increasingly tachycardic, tachypneic, and hypoxemic, and was transferred to the ICU on May 9th with worsening renal function and hypotension. His respiratory status was tenuous on high-flow oxygen. His family wished to see him, but they had been exposed to COVID-19 and face-to-face visitation was not possible.

Palliative care was consulted, and a telehealth family meeting was held with the PCT using the family’s smart phone to offer support and discuss who he was as a person, his condition and care goals. His family again described their distress about the absence of touch and caring they perceived at Hospital A. They shared his legacy including his strong Christian faith, his years of work with Veteran Service Organizations and his love of restoring old Harley motorcycles. He was a tinkerer who could fix anything, and an avid gardener and fisherman who took great pride in tying and designing his own flies. He loved to regale anyone who would listen with tall tales and could keep an audience spellbound. He especially missed his black Labrador who had been his close companion since he was a puppy, but who had died 6 months ago. His family believed he would not want to suffer in the hospital and that he would want to transition to a comfort measures only treatment plan. They were heartbroken not to be able to visit him in person, but willing to allow the PCT to set up video visits using a tablet computer at Mr. L’s bedside and the family’s own devices. Roles and responsibilities were discussed in advance of the bedside video visits. Each staff member was intentional about offering connection, compassionate care and touch, and checking back with family members to ensure their needs were being met. The PCT nurse coordinator (A.F.) arranged for the tablet to be brought to the patient’s room, scheduled times for each call, and obtained contact information for family members. The coordinator prepared the family regarding the patient’s condition and what they might see in the ICU room. The chaplain, ICU nurse practitioner, and bedside nurse donned PPE and established a video link with each family member using the tablet. The chaplain sang hymns, read his favorite Bible verses, and prayed with them. The nurse ensured that Mr. L’s physical symptoms were being addressed, and the nurse practitioner held the tablet so family could see the patient and the chaplain’s spiritual care interventions and answer medical questions as needed.

Family and staff debriefs were used to evaluate the success of the palliative care telehealth program. The PCT nurse coordinator debriefed with the family following the FaceTime visits. They were very pleased with how things went. Family were told that staff would continue to say their names to him, talk with him about family memories they wished to have shared with him, and ensure he was touched regularly. Although the circumstances were very sad, his family felt connected with him in his final hours and were able to say their goodbyes. In a staff debrief, participants found the experience surprisingly intimate and rewarding.

### Evaluate and Feedback Progress

This quality improvement project accomplished 2 positive outcomes: the successful deployment of video telehealth technology for inpatient EOL care; and the preservation of the intimacy and communication that is essential to palliative care. We found that implementing palliative care telehealth for inpatients involved building the infrastructure through protocol development, training, and physical infrastructure; and engaging the entire care team and family to explore non-traditional ways to visit with the patient.

A second consideration was attention to rapport and trust building. We found that adjustments for communication by video were critical, in line with the reported experience of other groups who have initiated palliative care telehealth consultations.^27,29^ These included identifying and planning care around family and provider needs including family perceptions of a perceived lack of human connection and physical touch; feeling that the patient’s basic needs were not being met; and the anguish of feeling socially isolated.

By listening to the family, their concerns and perspectives, we found ways to adapt the video visits to be more human and personalized. This included demonstrating human touch and caring at the bedside during the video calls, coaching family as to what they would see, inquiring about legacy issues and other specific needs including spiritual care, and incorporating these into the video visit. During and after the video calls, bedside staff used this guidance from family to speak to the patient about specific family members, family stories, hobbies/interests, offer music/entertainment the patient enjoyed, and offer spiritual care tailored to the patient’s needs. Staff performing bedside care reported a deeper connection and recognition of the person in the bed. His family valued the ability to see and talk with the patient virtually, and the opportunity to engage in life review and share stories of his legacy with healthcare staff. They expressed their gratitude in knowing the patient was being treated as a person.

In the end, we found that the technological aspects of care receded into the background and the human aspects remained visible. And, we were fortunate that the technology worked well.

### Modify/Extend Initial Action Plan as Needed

Given how much the incorporation of specific person-centered actions, storytelling and touch added to the experience for family and staff, we adapted our processes and trainings to formally include these components (Table 3). As the number of COVID-19 patients has decreased in our region we are aware that subsequent waves of infections may yet occur, and it is imperative to maintain and improve our palliative care telehealth program. We continue to engage with stakeholders throughout the facility to inform and educate them about our virtual palliative care model adaptations, and how we can assist them to incorporate technology into EOL-specific care. This involves on-the-spot and advance COVID-specific training of bedside nurses on EOL care, palliative care communication strategies, utilization of telehealth technology; facility-wide training in the use of our SOP; and meetings with medical directors and nurse managers of COVID-care and intensive care units regarding how to access the tablets and modifications in palliative care services during the pandemic.

## Conclusion

The pandemic was a powerful motivator for systemic change that propelled us to find new ways to provide caring and connectedness for patients at the EOL and their families. Our experience shows that although technology does not replace face-to-face encounters, it can offer meaningful connection. Redefining the traditional Palliative Care model required humility and the courage to embrace change. We are learning to live with the fear that technology will fail and that some families, patients and staff will be disappointed if the virtual care alternative is not “as good as” the “gold standard,” and are giving ourselves permission to make mistakes while we learn a new care model.

By conceptualizing this work as a business model innovation and identifying the processes necessary to deliver personalized virtual palliative care within a quality improvement framework, we hope to provide others with a useful template for reinventing palliative care service delivery in other care systems. While we recognize that the applicability of our work to other care settings and patients may be limited by the focus on a single case at one VA medical center, we extended its generalizability by grounding our approach in broadly accepted principles in business model innovation and quality improvement. In the spirit of learning together, we offer a few “take-home messages” in Table 5. We hope that the model we developed, and the lessons learned will be durable and positive outcomes from the pandemic.

**Table 5.** Take Home Messages.

The technology is imperfect; have a backup plan (probably the telephone)
Video connections will not work well for everyone; be prepared to accept that not everyone’s needs will be fully met. Most people will appreciate that you tried
“Don’t let the perfect be the enemy of the good”
Be flexible, creative and use the tools that are available: if you cannot get tablets, phones in every room are better than nothing. Other alternatives: Vocera devices; baby monitors; their personal smart phones to establish a connection
When starting a palliative care telehealth project, develop a business plan and use a rapid-cycle quality improvement approach
Do not wait for someone else to get things started. Start small, but do start

## Supplemental Material

Supplemental Material, Supplement_JHPM - Reinventing Palliative Care Delivery in the Era of COVID-19: How Telemedicine Can Support End of Life Care Click here for additional data file. Supplemental Material, Supplement_JHPM for Reinventing Palliative Care Delivery in the Era of COVID-19: How Telemedicine Can Support End of Life Care by Katherine C. Ritchey, Alice Foy, Erin McArdel and David A. Gruenewald in American Journal of Hospice and Palliative Medicine®
