# Supplementary material for: Reinventing Palliative Care Delivery in the Era of COVID-19: How Telemedicine Can Support End of Life Care
Source: Am J Hosp Palliat Care. 2020 Aug 7;37(11):992–7. doi: 10.1177/1049909120948235 (PMC7488822; doi:10.1177/1049909120948235)
Supplement: Supplemental Material, Supplement_JHPM - Reinventing Palliative Care Delivery in the Era of COVID-19: How Telemedicine Can Support End of Life Care [file Supplement_JHPM.pdf]

Supplemental Table 1: Example Standard Operating Procedure for Tablets in Inpatient Palliative Care Consultation and Family Visitation.

RESPONSIBILITIES:

1. Staff using tablets for consultations and family visitations shall be mindful to minimize additional burden on front-line clinical staff and ensure that PPE use is not increased due to tablet usage, by ensuring that tablets are deployed to the bedside in association with trips into the patient's room for other care that is being given.
2. PCT staff will be responsible for safe storage and deployment of tablets for palliative care consultations and family visitations and will account for each device daily.
3. Clinical staff from inpatient units or the emergency department who request access to tablets will be responsible for checking out tablets held by the palliative care staff and returning them to palliative care staff.
4. All staff using tablets will ensure the security and cleanliness of each tablet they use for clinical care. Staff will ensure that patients are logged out of their personal accounts on the device after use and will be responsible for cleaning tablets before and after each clinical care use, to the specifications described below

EQUIPMENT:

1. Patients: Stock tablet, with protective case. Each tablet is individually named, with individual ID and passcode protected
2. PCT or other clinical staff: workstation, laptop, or tablet equipped to provide CVT, including webcam and microphone
3. Family: Family-owned device equipped with camera, microphone, Wi-Fi data plan, and VA Video Connect (VVC) or alternative VA-approved option for CVT

PROCEDURE*:

1. Storage and Tracking: Tablets will be kept in palliative care team office in locked drawer.
    a. Non-PCT staff wishing to access a tablet will place consult request to PCT to document use of device in the electronic health record.
    b. Staff will check out equipment from storage by entering their name, service, contact info, date/time checked out, and date/time returned on a written logbook in the PCT office.
2. Consent: CVT will be offered to the patient (if able to consent/participate) and/or DPOA/LNOK/family. Risks/benefits of CVT will be explained, and consent documented in the clinical record if consent to use CVT is granted. Patient/family may opt out at any time.
3. Creation of CVT encounter/appointment: For clinical consultations, PCT or other staff with completed telehealth training will create a CVT encounter using Virtual Care Manager (VCM) with link to the Virtual Medical Room (VMR). Staff will Email link to the tablet to be deployed to the bedside with unique identifying email/ID. For family visitations, email will be sent to family representative at the family's email address.
4. Deployment of tablet to bedside: PCT or clinical staff will take tablet to Veteran's room. Encounter will be planned to align with other bedside patient care provided by front line healthcare provider (HCP).

a. Before patient use, PCT or trained staff will ensure that tablet devices are clean, safe and ready for each encounter by performing thorough hand hygiene (handwashing or use of alcohol-based hand sanitizer), donning a clean pair of gloves, and wiping all surfaces of the tablet with purple-topped Sani-cloth wipes ensuring this surface remains wet for two minutes. After observing wet time, staff will remove gloves and perform hand hygiene.
b. Staff will establish a Wi-Fi connection and open CVT encounter on the tablet, then place tablet in clear plastic bag (Ziploc equivalent) and seal the bag.
c. PCT or trained staff will give bagged tablet to donned HCP who will give it to patient (tablet stays in bag).
d. After patient use, HCP will stand just inside the door of the patient's room and open the plastic bag with PCT or trained staff at the door just outside the room. Having performed hand hygiene and donned clean gloves, PCT or trained staff will remove the tablet from the bag held by HCP, taking care not to touch the bag. HCP will dispose of the bag in a trash receptacle inside the patient's room. PCT or trained staff will place the tablet on a clean paper towel, de-glove, perform hand hygiene, re-glove, then wipe all surfaces of the tablet with purple-topped Sani-cloth wipes ensuring this surface remains wet for two minutes. After observing wet time, dispose of paper towel in trash receptacle, remove gloves and perform hand hygiene.
e. PCT or trained staff will return tablet to palliative care office located in bldg 100, 7C-102 and log the device as returned in the logbook. PCT will complete non-visit consult after tablet is returned.

Tablet Cleaning Procedure for Patients with C. Difficile Colitis:

1. Staff will follow all procedures as described above, with the following modifications:
   a. After patient use, tablet will be sanitized with bleach wipes, and staff will perform hand hygiene steps with handwashing (not alcohol-based hand sanitizer).

*NOTE: Procedures for creation of patient appointment and encounter are specific to VA Video Connect system. Procedures for other software systems will differ

ABBREVIATIONS:
PPE – personal protective equipment
PCT – palliative care team
CVT – clinical video telehealth
DPOA – durable power of attorney
LNOK – living next-of-kin
HCP – healthcare provider
